# Feasibility of single‐port retroperitoneoscopic adrenalectomy in dogs

**DOI:** 10.1111/vsu.12789

**Published:** 2018-04-26

**Authors:** Jonghyeok Ko, Junemoe Jeong, Sungin Lee, Hyunglak Son, Oh‐Kyeong Kweon, Wan Hee Kim

**Affiliations:** ^1^ Department of Veterinary Clinical Science, College of Veterinary Medicine and Research, Institute for Veterinary Science Seoul National University Seoul Republic of Korea

## Abstract

**Objective:**

To evaluate the feasibility of single‐port retroperitoneoscopic adrenalectomy (SPRA) in dogs.

**Study Design:**

A pilot experimental study.

**Animals:**

Eight healthy beagle dogs.

**Methods:**

SPRA was performed on the left and right sides (4 dogs each). Resection of the adrenal gland was performed through a SILS port using a retroperitoneal approach. Operative time was defined from skin incision to the completion of skin suture. Postoperative pain was evaluated by using 3 pain scores. Integrity of the adrenal gland capsule was evaluated by histologic assessment.

**Results:**

Mean time taken to complete the SPRA was 44.1 minutes (range, 37‐51) and was significantly longer on the right side than on the left side (*P* < .05). There were no complications intraoperatively or during 14 days of postoperative monitoring. The adrenal gland capsule was found to be injured in 3 of the 8 dogs by histologic assessment.

**Conclusion:**

This is the first report of SPRA in the veterinary literature. With this technique it is possible to perform adrenalectomy with some risk of capsule penetration and with excellent visibility.

**Clinical significance:**

This study suggests that SPRA is feasible and can be used to resect small adrenal tumors with minimal complications.

## 1 INTRODUCTION

Laparoscopic adrenalectomy is a minimally invasive procedure that is becoming an alternative to conventional veterinary surgery.^1^, ^2^, ^3^, ^4^, ^5^ Previous reports have documented that a laparoscopic adrenalectomy (90 minutes) was shorter than that required for open adrenalectomy (120 minutes).^1^ Moreover, some reports indicate that modified laparoscopic adrenalectomy in dogs has advantages over laparoscopic adrenalectomy.^2^, ^3^ When laparoscopic adrenalectomy was performed in lateral recumbency, as described by Jiménez Peláez et al,^3^ the surgery was feasible and without intraoperative complications. Laparoscopic adrenalectomy performed in sternal recumbency also has advantages, allowing gravitational displacement of the abdominal organs, better visualization of the adrenal gland, and a shorter surgical time than conventional laparoscopy or open adrenalectomy. Mean surgical time was reported to be 78.7 minutes.^2^ However, both methods require retraction of the abdominal organs.^2^, ^3^

In human medicine, operative times for laparoscopic adrenalectomy have not shortened since the procedure was first performed in 1992.^6^ Retroperitoneoscopic adrenalectomy was devised in 1994 to address this issue.^6^, ^7^, ^8^, ^9^ After standardization of the procedure, it has been reported that retroperitoneoscopic adrenalectomy had the advantages of a shorter operative time, less blood loss, and less pain compared with transperitoneal laparoscopic adrenalectomy. It has been suggested that these advantages are due to minimal organ retraction allowing direct access to the adrenal gland.^6^, ^10^ Recently, surgical procedures performed through a single port have become popular in human medicine because they afford better patient comfort, superior cosmetic results, less pain, and a more rapid functional recovery.^11^, ^12^, ^13^, ^14^ Single‐port retroperitoneoscopic adrenalectomy (SPRA) is a minimally invasive surgery and has the additional advantages of its use of single‐port access and the ability to use a retroperitoneal approach.^15^, ^16^, ^17^, ^18^, ^19^

Although retroperitoneal access to the adrenal gland has been documented,^20^ retroperitoneoscopy has been only mentioned rarely in the veterinary literature.^5^ In our previous study describing the development of retroperitoneal access and the evaluation of working space and anatomy in 6 dogs, we found that the retroperitoneoscopic approach was viable and allowed good visualization.^21^ A single‐port retroperitoneal approach to adrenal gland resection has the possibility of a better outcome than that achieved by using several conventional surgical methods. In this study, we used a modified posterior retroperitoneoscopic adrenalectomy technique developed for man,^22^, ^23^, ^24^ taking into account the differences between human and canine anatomy,^25^, ^26^ to try to improve the outcome of adrenalectomy in dogs. The objectives of this study were to determine whether retroperitoneal laparoscopy is a feasible approach to the adrenal gland, allowing direct access for adrenalectomy and that this approach may be performed with minimal complications, post operative pain or damage to the adrenal gland.

## 2 MATERIALS AND METHODS

Eight healthy beagle dogs (mean body weight 11.3 ± 1.0 kg, mean Body Condition Score 5.2 ± 0.9) were allocated to undergo either right‐sided (n = 4) or left‐sided (n = 4) adrenalectomy. All dogs received premedication with acepromazine 0.01 mg/kg IV, tramadol 5 mg/kg IV, meloxicam 0.2 mg/kg subcutaneously, and cefazolin 22 mg/kg IV. Alfaxalone 2 mg/kg IV was used to induce anesthesia, which was maintained by using inhalational isoflurane with 100% oxygen. All SPRA procedures were performed by 1 surgeon (JK). Operative time was defined as the time from skin incision to the completion of skin sutures. Port placement time was measured from the time of first incision to that of a SILS port insertion. The study was approved by our institution's animal care and use committee.

### 2.1 Single‐port retroperitoneoscopy

Dogs were positioned in sternal recumbency without abdominal support with 3 cushions and self‐adhesive tape, as described by Naan et al.^2^ The lateral aspect of the hemithorax and hemiabdomen was clipped from the level of the 11th thoracic vertebra to the level of the seventh lumbar vertebra for aseptic surgery. To access the retroperitoneal space, one needs to cut through 6 layers. Palpating the transverse process (left, L2 and L3; right, T13 and L1), a 3‐cm incision was made between the transverse processes of each palpated vertebra. Electrocautery was used for hemostasis to prevent hemorrhage after the skin incision was made with a scalpel. Blunt dissection with Metzenbaum scissors, with the index finger positioned below the epaxial muscle, was used to access the retroperitoneal space through the muscle structures (external oblique, internal oblique, and transverse abdominis). The ability to palpate the dorsal aspect of the kidney indicated successful entry into the retroperitoneal space after dissection of the thoracolumbar fascia. A SILS port (Covidien, New Haven, Connecticut) was placed by using a stay suture for retraction of the skin and muscles. After placing three 5‐mm cannulas through the SILS port for triangulation of the laparoscopic instruments, the retroperitoneal space was investigated by using a 5‐mm, 0° telescope (Karl Storz, Tuttlingen, Germany). When the retroperitoneal space was visualized, pneumoretroperitoneum was induced by using an insufflator (Karl Storz) at a pressure of 5 mmHg.

### 2.2 Left adrenalectomy (L2‐3)

**Figure 1:**
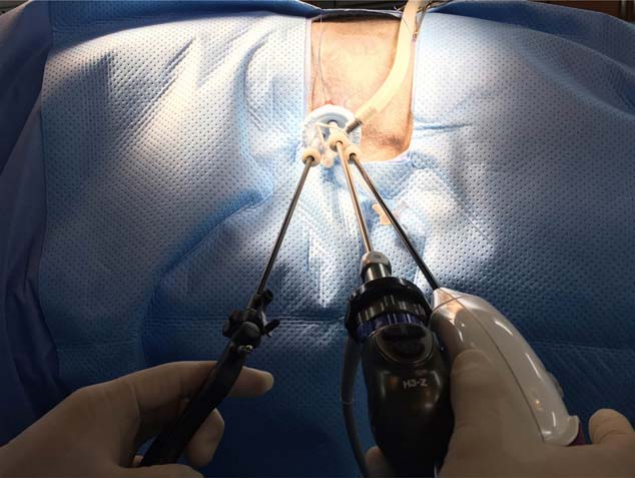
Single‐port placement via a SILS port in left retroperitoneoscopic adrenalectomy. Each of the instruments was introduced through 3 cannulas

**Figure 2:**
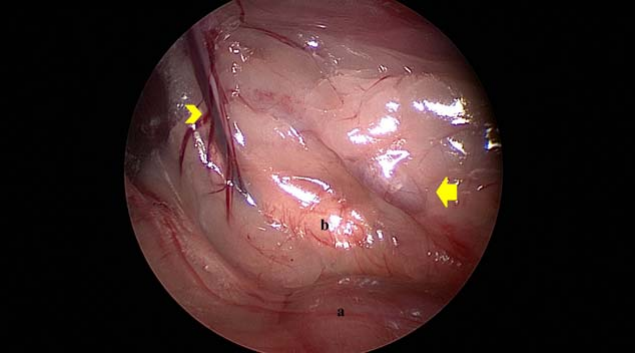
Left retroperitoneoscopic approach. After dissection of fatty tissue in the retroperitoneal space, the retroperitoneal organs could be seen directly: left kidney (a), left adrenal gland (b). The arrow indicates renal vessels. Arrowhead indicates the phrenicoabdominal vein

**Figure 3:**
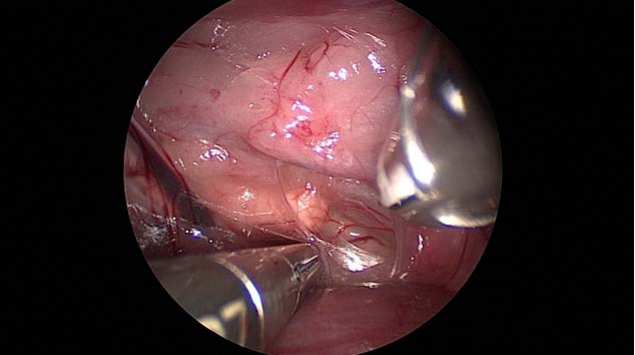
Left retroperitoneoscopic adrenalectomy. The caudal aspect of the left adrenal gland was dissected first. The branches of the phrenicoabdominal artery flow into the adrenal gland

**Figure 4:**
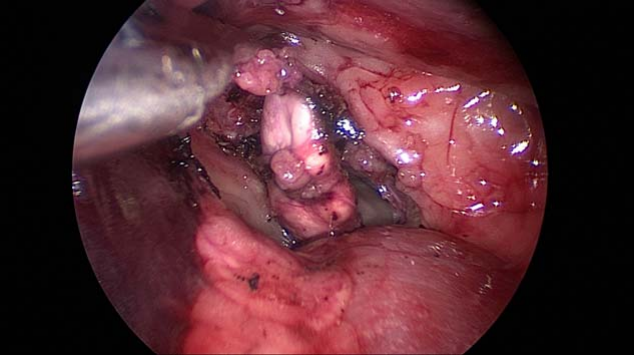
The retroperitoneal space before removal of the left adrenal gland. The gland was grasped with a Kelly forceps. To prevent damage to the adrenal gland, the periadrenal tissue was gripped for retraction of the gland

The telescope was introduced into the retroperitoneal space via the middle cannula. The other cannulas were used to insert a Kelly forceps (Karl Storz) and a vessel‐sealing device (LigaSure dolphin tip 5‐mm sealer and divider connected to a LigaSure or Force Triad generator; Covidien, Mansfield, Massachusetts), as displayed in Figure 1. Initially, the fascia and fat tissue were dissected delicately by using the telescope, resulting in exposure of the retroperitoneal structures (ie, the kidney, renal vessels, phrenicoabdominal vein, and adrenal gland; Figure 2). Taking care not to damage the left renal vein, the surgeon first dissected the left periadrenal gland tissue at the caudal aspect and then at the medial aspect for circumferential dissection. This enabled the periadrenal tissue to be held steady while the adrenal gland was retracted (Figure 3). During dissection of the caudal and medial aspects, the phrenicoabdominal vein running through the middle of the adrenal gland was isolated by dissection of the adjacent tissue and was transected. The cranial pole of the gland was surrounded by the retroperitoneum, to which the visceral adrenal capsule was attached. Because of these anatomic features, the cranial retroperitoneum, including the cranial surface of the gland, was resected together with the visceral adrenal capsule. After dissecting the cranial aspect of the adrenal gland, the ventral aspect was dissected. The excised adrenal gland was removed through the incision line (Figure 4).

### 2.3 Right adrenalectomy (T13‐L1)

**Figure 5:**
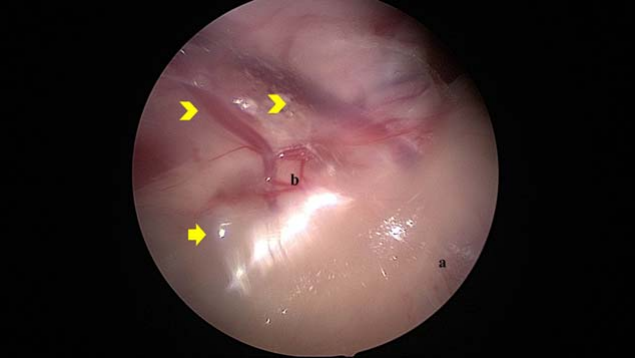
Right retroperitoneoscopic approach. Unlike in the left retroperitoneoscopic approach, the phrenicoabdominal artery is distinguished clearly: right kidney (a), right adrenal gland (b). Arrow indicates renal vessels. Arrowheads indicate phrenicoabdominal vessels

**Figure 6:**
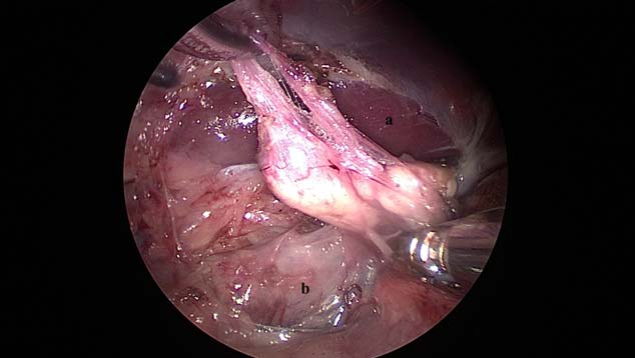
Right retroperitoneoscopic adrenalectomy. The LigaSure is used to dissect the capsule of the gland from the caudal vena cava. Epaxial muscle (a), caudal vena cava (b)

**Figure 7:**
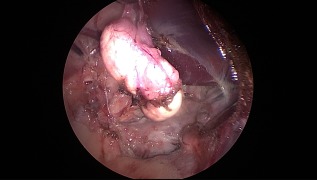
View of the right adrenal gland after all aspects were dissected. None of the injuries were on the caudal vena cava. Hemorrhage seldom occurred in the retroperitoneal space during resection

After placement of the SILS port, the fascia and fat tissue were dissected under telescopic guidance via the middle cannula of the port (Figure 5). The right renal vessels were located laterally and were connected to the right kidney. The phrenicoabdominal artery and vein, which are located at the region cranial to the right renal vessels, were transected at the point proximal to the branches, many of which extended to the periadrenal tissues. The distance between the caudal pole of the right adrenal gland and the renal vein was greater than that between the left adrenal gland and the renal vein. After dissection of the caudal aspect of the periadrenal tissue, this tissue was grasped with the Kelly forceps for retraction of the adrenal gland. The medial and cranial aspects of the adrenal gland were dissected, thus exposing the caudal vena cava on the ventral surface of the gland. Upon retraction of the adrenal gland in the dorsal direction, the gland was separated from the caudal vena cava but remained connected to this vessel by the gland capsule (Figure 6). After dissection from the caudal vena cava, the right adrenal gland was resected completely (Figure 7).

### 2.4 Closure

External oblique, internal oblique, and transverse abdominis muscles were sutured together in 1 line by using a simple continuous pattern (PDS II 3‐0; Ethicon, Cornelia, Georgia). Subcutaneous suturing was completed by using a simple continuous pattern (PDS II 4‐0; Ethicon), and the skin was sutured by using a simple interrupted pattern (Nylon 4‐0; Ailee, Busan, Republic of Korea).

### 2.5 Postoperative care

At the time the incision was closed, local anesthetic was infiltrated to the operative area by using a mixture of bupivacaine 0.2 mg/kg and lidocaine 1.6 mg/kg. An oral antibiotic (cephalexin 22 mg/kg twice daily) and oral analgesics (tramadol 5 mg/kg twice daily, meloxicam 0.1 mg/kg once daily) were administered for 7 days after SPRA.

### 2.6 Assessment of postoperative pain

Pain was assessed after recovery from anesthesia and on postoperative days 3, 7, and 14. Three scoring methods were used to assess the pain associated with SPRA. The first method was the Numerical Rating Scale, which scores items such as demeanor (0‐4), movement (0‐2), appearance (0‐3), behavior (0‐3), interactive behavior (0‐3), vocalization (0‐3), heart rate (0‐3), and respiration rate (0‐3).^27^, ^28^, ^29^, ^30^, ^31^ The maximum possible score is 24, with 0‐8 indicating mild pain, 9‐16 indicating moderate pain, and 17‐24 indicating severe pain. The second method was the University of Melbourne Pain Scale, which scores physiologic data (0‐11), response to palpation (0‐3), activity (0‐3), mental status (0‐3), posture (0‐4), and vocalization (0‐3).^28^, ^32^ This scale also scores the severity of pain as mild (0‐9), moderate (10‐18), or severe (19‐27). The third method was the Colorado State University Veterinary Medical Center Canine Acute Pain Scale, which scores psychological and behavioral items, response to palpation, and body tension.^28^ Each of the 3 dimensions is measured on a 4‐point pain scale (0, none; 1, mild; 2, moderate; 3, severe). One designated investigator assessed the postoperative pain score for all 3 methods in a blinded manner. Mean pain scores above moderate (>9, >10, and >2, respectively) were used to judge the requirement for additional analgesia.

### 2.7 Histological assessments

After measurement of the thickness of the adrenal glands at the cranial and caudal poles, excised adrenal glands were stored in specimen cups containing 10% neutral formalin. Tissue fixation time was more than 48 h. Fixed samples were dehydrated before being embedded in paraffin. Samples were divided at this time into 4 pieces for precise evaluation of damage to the capsule. Paraffin blocks were sliced in 5‐μm‐thick sections. All slides were stained with hematoxylin‐eosin. The capsule of each excised adrenal gland was assessed for surgical damage.

### 2.8 Statistical analysis

All data were analyzed in SPSS Statistics version 23 (IBM, Armonk, New York). Statistical significance of differences in operation and port placement times between the left and right approaches was evaluated by using the Student *t* test.

## 3 RESULTS

### 3.1 Operative time

Mean ± SD operative time was 44.1 ± 6.1 minutes (range, 37‐51) for retroperitoneoscopic adrenalectomy, 49.3 ± 1.5 minutes (range, 48‐51) for right SPRA, and 38.5 ± 1.29 minutes (range, 37‐40) for left SPRA (Table 1). Operative time for right SPRA was significantly longer than that for left SPRA (*P* < .05).^2^, ^3^ The difference in mean port placement time (9.8 ± 1.36 minutes; range, 8‐12 min) between the left and right approaches was not significant (*P* > .05).

**Table 1:** Surgical details of retroperitoneoscopic adrenalectomies in this study

Dog	Site	Operation time, min	Cranial pole, mm	Caudal pole, mm
1	Left	40	10	11
2	Left	37	7	8
3	Left	38	11	7
4	Left	39	8	7
5	Right	48	10	9
6	Right	50	10	5
7	Right	48	12	8
8	Right	51	12	6

### 3.2 Outcome

All SILS ports were placed successfully. Without retraction of other organs, a sufficient surgical view was secured, and the adrenal gland was approached directly and easily excised. Only periadrenal tissues were manipulated for the dissection of adrenal glands. Conversion of adrenalectomy to laparotomy was not required, and no severe bleeding events occurred. There were no instances of injury to adjacent tissue. Most dogs adopted a standing position and were walking within 30 minutes of extubation. There were no postoperative complications such as port site hemorrhage, hematoma, dehiscence, or seroma.

### 3.3 Assessment of postoperative pain

Mean scores on the Numerical Rating Scale, the University of Melbourne Pain Scale, and the Colorado State University Veterinary Medical Center Canine Acute Pain Scale were <9, <10, and <2, respectively (5/24, 6.5/27, and 0.75/4, respectively, for the left side, and 5.75/24, 7/27, and 0.92/4, respectively, for the right side), indicating that postoperative pain after recovery from anesthesia was mild and that the administered analgesics were adequate for pain control. Lower pain scores were recorded for all dogs on days 3, 7, and 14 after surgery (Table 2).

**Table 2:** Postoperative pain assessment after retroperitoneoscopic adrenalectomy^a^

Scale (range)	Side	IP	PD3	PD7	PD14
NRS (0‐24)	Left	5 (1.83)	2.26 (0.96)	1.25 (0.5)	1 (0)
	Right	5.75 (1.26)	2.5 (0.58)	1.25 (0.5)	1 (0)
UMPS (0‐27)	Left	6.5 (2.38)	4 (2.45)	2.5 (1)	2 (0)
	Right	7 (0.82)	4.75 (0.5)	2.5 (1)	2 (0)
CSUPS (0‐4)	Left	0.75 (0.5)	0.58 (0.32)	0.08 (0.17)	0 (0)
	Right	0.92 (0.92)	0.67 (0.32)	0.16 (0.19)	0 (0)

CSUPS, Colorado State University Veterinary Medical Center Canine Acute Pain Scale; IP, immediately postoperative; NRS, Numerical Rating Scale; PD, postoperative day; UMPS, University of Melbourne Pain Scale;

a Values are mean (SD).

### 3.4 Histological assessment

Mean thickness of the cranial pole was 1 ± 0.18 cm (range, 0.7‐1.2), and that of the caudal pole was 0.76 ± 0.18 cm (range, 0.5‐1.1; Table 1). Three adrenal glands were found to have sustained damage to the capsule on slides stained with hematoxylin‐eosin. The injuries were limited to small defects in the capsule. The adrenal parenchyma, including the cortex and medulla, was unaffected in all cases.

## 4 DISCUSSION

Both right‐sided and left‐sided adrenalectomy procedures were feasible by using SPRA without the requirement for additional organ retraction in this study. Right‐sided and left‐sided adrenalectomy port placement time was similar, but operative time during right‐sided adrenalectomy was longer than the time during left‐sided adrenalectomy. Additional analgesics and conversion to laparotomy were not required intraoperatively. All dogs recovered rapidly from the surgery to ambulatory status without any complications. Dogs with mild pain were not given additional analgesics, resulting in low pain scores for 14 days after the surgery. Therefore, the postoperative analgesic protocol was adequate for pain management in this study. SPRA provides direct access to and a clear view of the adrenal gland, which are not possible in transperitoneal laparoscopy.

### 4.1 Retroperitoneoscopic approach for adrenalectomy

The human retroperitoneum can be divided into 3 anatomic regions, anterior perirenal, perirenal, and posterior perirenal space. Several organs are in the anterior perirenal space, which is between the peritoneum and anterior perirenal fascia, such as the colon, duodenum and pancreas, whereas they are peritoneal organs in dogs. On the other hand, the perirenal space and posterior perirenal space behind the anterior perirenal space contain the kidneys, adrenal glands, upper ureters, fat, and muscles.^33^ Posterior retroperitoneoscopic adrenalectomy is preferred in man^10^, ^22^, ^24^, ^34^, ^35^, ^36^ because it does not require entry into the peritoneal cavity, and, unlike transperitoneal laparoscopic surgery, it does not create the risk of postoperative adhesions.^22^, ^24^ In addition, there is no requirement to reposition patients as there is for bilateral adrenalectomy in a sternal position. Unlike in man, the anatomic structure of the dog cannot be divided into several regions. As the retroperitoneal area in the dog is similar in form to that of the human posterior retroperitoneum, including the perirenal and posterior perirenal space, we considered that this approach would be feasible in dogs.^25^

For retroperitoneoscopic adrenalectomy, the landmark for access into the retroperitoneal space is the tip of the 12th rib in man, but the equivalent landmark has not been reported in dogs.^34^, ^35^, ^36^, ^37^, ^38^, ^39^ We believed that it was possible to approach the retroperitoneum by using the transverse processes of a vertebra as the landmark. Correct initial placement of the port is important when accessing the retroperitoneal space. When the retroperitoneal approach fails during surgery, pneumoperitoneum makes it difficult to reapproach the retroperitoneum because of its effect on the location of the retroperitoneal organs. We used an approach that entailed blind palpation with a finger and blunt dissection for port placement at the level of T13 and L3, which is similar to the approach for open retroperitoneal adrenalectomy reported in both man and dog.^6^, ^8^, ^20^ The left and right adrenal glands are located at L2 and T13, respectively. Therefore, L2‐L3 for left adrenalectomy and T13‐L1 for right adrenalectomy were considered appropriate locations from which to make an approach. After the external oblique, internal oblique, and transverse abdominis muscles were dissected, the thoracolumbar fascia forming the parietal retroperitoneum was palpated. Penetration of this fascia led to palpation of the kidneys, indicating entry to the retroperitoneum. To prevent leakage of CO_2_ in our previous study,^21^ we used a blunt‐tip trocar with a balloon, as used in man, because the retroperitoneal approach is an open‐entry technique.^8^, ^22^, ^34^, ^38^, ^39^ In man, 2 additional ports are placed after the trocar to approach the adrenal gland. Unlike in man, setting of the landmarks for additional ports is difficult in dogs because the ventral part of the retroperitoneum is not attached to the transverse abdominis muscle; insertion of additional ports is both challenging and time consuming. Therefore, we elected to use a single‐port approach through a small 3‐cm incision in this study. Because the flexible single port can secure the thoracolumbar fascia, which is in the deepest part of the retroperitoneum, it was possible to maintain inflation of the retroperitoneal space without leakage of gas, as occurs with a blunt‐tip trocar. Moreover, another potential advantage of single‐port surgery is reduction in operative time that would be required to place additional ports.

### 4.2 Anatomic structures

With this approach, we observed adipose tissues similar to those of Gerota's fascia in man.^22^ This tissue can be removed simply under telescope guidance just as in man, and, upon dissection, the adipose tissues surrounding the various organs in the retroperitoneoscopic area were visible. After the adipose tissues were dissected, the contours of each organ could be precisely viewed. The first landmark to identify is the kidney, which is found in the ventrocaudal view. The adrenal gland was located by dissecting the cranial side of the renal vessel where the vessel runs in the mediodorsal direction from the kidneys. There was no need to pull or manipulate the other organs while isolating the adrenal gland. The second landmark to identify when locating the adrenal gland is the phrenicoabdominal vein, which runs through the middle part of the adrenal gland between the cranial and caudal poles of the gland. In the right‐sided approach, the phrenicoabdominal artery is clearly distinguished; however, this is not true for the left‐sided approach. For the transection, a vessel‐sealing device was used to separate the tissues around the adrenal gland. The epaxial muscle was found in the mediodorsal area; after resection of the adrenal gland, the caudal vena cava, to which the capsule of the gland was connected, was found on the ventral aspect of the gland.

### 4.3 Dissection

For a right adrenalectomy, retroperitoneoscopy has the advantage of providing good visibility of the capsule connected to the caudal vena cava, which can reduce the risk of damage to this vessel. Unlike on the right side, the ventral aspect of the left adrenal gland is connected to the peritoneum and can be dissected easily. The dissection took a shorter time for left adrenalectomy than for right adrenalectomy. As reported in man,^6^, ^39^ neither right nor left retroperitoneoscopic adrenalectomy requires preparation of the abdominal organs, which might decrease complications such as pancreatitis, intestinal obstruction, and adhesions.

The cranial part of each adrenal gland is attached to the peritoneum. The peritoneum should be dissected completely so that the cranial side can be examined thoroughly before resection. Poor visibility may cause bleeding and make complete resection impossible. The retroperitoneal and peritoneal cavities may be connected because a hole is created in the peritoneum. However, even if the cavities are connected, the view is not obstructed whether left adrenalectomy or right adrenalectomy is performed because inflation of the peritoneal cavity does not put pressure on the retroperitoneal cavity.

### 4.4 Manipulation of instruments

When SPRA was first performed in man, the large vessels were ligated by using an endoscopic hemoclip.^6^, ^7^, ^8^, ^38^, ^39^ Use of the LigaSure is regarded as a better method for sealing and dividing vessels because it reduces the operative time.^10^, ^34^ In man, the adrenal gland is connected to the inferior, middle, and superior adrenal arteries and veins.^22^, ^33^, ^37^ However, in dogs, the adrenal artery is connected to a number of branches of the phrenicoabdominal artery; thus, dissection of the periadrenal tissues is likely to cause arterial bleeding.^26^ This problem can be easily overcome if the LigaSure is used for dissection and hemostasis.

The disadvantage of using a single port is that the instruments may collide with each other, which is common in the small working space involved when the retroperitoneoscopic approach is used. However, the SILS port consists of flexible material that allows a large degree of freedom to manipulate laparoscopic instruments. Furthermore, the resection might not be difficult for a surgeon with laparoscopic experience. Although we used nonarticulated surgical instruments, this surgery may be even more effective if performed with articulated instruments, which can be manipulated more comfortably.

### 4.5 Outcome

In this study, there were no complications such as subcutaneous emphysema, hematoma, hernia, dehiscence, or hemorrhage, which may be reported after this type of surgical procedure.^22^, ^35^ Substantial hemorrhage, which is a possible complication during the surgery, will not occur if the renal vessels, aorta, and caudal vena cava are securely separated in the approach and during resection of the adrenal gland. If severe bleeding occurs, conversion to open surgery would be required, but this should not affect the outcome for the patient.^40^

### 4.6 Postoperative pain

In contrast with multiport laparoscopic surgery, some reports in man suggest that SPRA is less painful, requires a shorter hospital stay, and has cosmetic benefits.^16^, ^17^, ^18^, ^19^ In one of these studies, visual analog pain scale scores suggested that pain after single‐port retroperitoneoscopic surgery was less severe than that after transperitoneal laparoscopic surgery.^18^ When we palpated the surgical site in the postoperative period, no dogs showed a marked pain response, such as guarding the wound site or aggressive behavior. In this study, a mild pain response was observed postoperatively in all dogs according to all 3 pain assessment methods. This indicates that pain levels were manageable. However, transient and mild pain was present in the short term.

### 4.7 Resected adrenal gland tissues

In man, the indications for posterior retroperitoneoscopic adrenalectomy and transperitoneal adrenalectomy are limited to benign tumors. Furthermore, a resectable adrenal gland size limit of 6 cm is specified.^22^ However, in dogs, a clinical study is required to determine the size limit for resection. If the 3‐cm incision used in the present study is taken as a guide, tumors up to 3 cm could be removed through the incision line, and SPRA may be limited to benign tumors (eg, incidentalomas and adenomas).

Damage to the capsule appeared mild in this study, although this may not be the case when excising an adrenal tumor. Spillage of tumor cells via a damaged capsule can occur in laparoscopic adrenalectomy, leading to peritoneal carcinosis.^1^, ^3^, ^6^, ^41^ Retroperitoneoscopic surgery might reduce the risk of tumor cells seeding to the abdominal organs (in comparison with transperitoneal laparoscopic adrenalectomy) because the retroperitoneal space is separated from the peritoneal space.

This study provides evidence that SPRA is feasible in dogs. However, this research has some limitations. First, our study did not include an investigation of clinical safety or any aspects of clinical application. Second, the study population did not include various sizes and breeds of dogs. Third, we did not compare retroperitoneoscopic adrenalectomy with other techniques used to perform adrenalectomy. Fourth, the number of dogs included in the study was too small to evaluate the outcome of SPRA. However, among the minimally invasive techniques that are used to perform adrenalectomy in dogs, SPRA may be preferable because, unlike conventional adrenalectomy, it is a direct approach that enables resection of the adrenal gland without any requirement to retract the other organs. In addition, postoperative pain levels were relatively mild after SPRA, which is beneficial in the clinical setting. Additional studies are required to compare the clinical benefits of SPRA with those of conventional surgical methods in dogs.

## CONFLICT OF INTEREST

The authors declare that there are no conflicts of interest related to this report.
